# Multicriteria Decision-Making in Diabetes Management and Decision Support: Systematic Review

**DOI:** 10.2196/47701

**Published:** 2024-02-01

**Authors:** Tahmineh Aldaghi, Jan Muzik

**Affiliations:** 1 Spin-off Companies and Research Results Commercialization Center First Faculty of Medicine Charles University Prague Czech Republic; 2 Department of Information and Communication Technologies in Medicine Faculty of Biomedical Engineering Czech Technical University Prague Czech Republic

**Keywords:** analytical hierarchy process, diabetes management, diabetes recognition, glucose management, multi-criteria decision making, technique for order of preference by similarity to ideal solution, decision support, diabetes, diabetic, glucose, blood sugar, review methodology, systematic review, decision making, self-management, digital health tool

## Abstract

**Background:**

Diabetes mellitus prevalence is increasing among adults and children around the world. Diabetes care is complex; examining the diet, type of medication, diabetes recognition, and willingness to use self-management tools are just a few of the challenges faced by diabetes clinicians who should make decisions about them. Making the appropriate decisions will reduce the cost of treatment, decrease the mortality rate of diabetes, and improve the life quality of patients with diabetes. Effective decision-making is within the realm of multicriteria decision-making (MCDM) techniques.

**Objective:**

The central objective of this study is to evaluate the effectiveness and applicability of MCDM methods and then introduce a novel categorization framework for their use in this field.

**Methods:**

The literature search was focused on publications from 2003 to 2023. Finally, by applying the PRISMA (Preferred Reporting Items for Systematic Reviews and Meta-Analyses) method, 63 articles were selected and examined.

**Results:**

The findings reveal that the use of MCDM methods in diabetes research can be categorized into 6 distinct groups: the selection of diabetes medications (19 publications), diabetes diagnosis (12 publications), meal recommendations (8 publications), diabetes management (14 publications), diabetes complication (7 publications), and estimation of diabetes prevalence (3 publications).

**Conclusions:**

Our review showed a significant portion of the MCDM literature on diabetes. The research highlights the benefits of using MCDM techniques, which are practical and effective for a variety of diabetes challenges.

## Introduction

### Overview

Diabetes mellitus is a chronic disease that is characterized by impaired insulin production and action [1]. According to the etiopathology of diabetes, the 3 most common clinical categories are distinguished: type 1 diabetes, type 2 diabetes (T2D), and gestational diabetes mellitus [2,3]. In recent decades, diabetes prevalence has increased in both adults and children around the world. By 2035, there will be an estimated 592 million people worldwide with diabetes [4]. By 2040, this number is expected to rise to 642 million [5], and by 2045, there will be 783.2 million cases of diabetes worldwide [2]. According to the global 2021 findings of the International Diabetes Federation (IDF), 537 million adults are living with diabetes, and 3 in 4 of them reside in low- and middle-income countries. In 2021, a total of 6.7 million people died of diabetes, equating to 1 death every 5 seconds. The expenditure on diabetes-related health care is at least US $966 billion, and it has increased up to 316% over the last 15 years [2].

Diabetes is a chronic condition requiring continuous medical care and patient education to prevent severe complications and long-term risks. Managing diabetes involves addressing various aspects of the patient’s health, including blood glucose monitoring, monitoring and managing carbohydrate intake, regular engagement in physical activity, and medication management. By understanding the disease’s nuances and recognizing when it might become severe, people can take steps to protect their well-being. Thus, faster diagnosis of diabetes and its potential complications is crucial for both patients and health care providers [6]. General practitioners faced a significant problem when diagnosing diabetes, partly because patients displayed a wide range of signs and symptoms. This complex clinical environment confused general practitioners and changed the diagnostic procedure into a multiobjective health care decision-making challenge [7].

In addition to making informed decisions about the patient's health, endocrinologists and general practitioners should carefully assess various factors, including lifestyle choices, dietary habits, daily physical activity levels, insulin requirements, and the patient’s willingness to embrace self-management technologies such as insulin pumps or pens, smart bracelets, continuous glucose monitoring, and mobile apps [8]. This comprehensive evaluation enables them to select the most appropriate treatment options. As an illustration, when it comes to managing hyperglycemia in patients with T2D, there is a diverse array of treatment options available. Currently, approximately 30 medications belonging to 9 distinct therapeutic categories have received approval for use, with ongoing research and development efforts yielding additional drugs and novel drug categories [9]. Due to the variety of options and guidelines from organizations such as the American Diabetes Association (ADA) [10], doctors often customize prescriptions using different doses and combinations for effective diabetes management [9]. The available medications vary in efficacy, safety, dosage, side effects, and cost. A lack of comparative information across these factors often leaves patients and physicians unable to make well-informed decisions [11]. The selection of diabetes medication presents itself as a multiobjective problem within the realm of health care decision-making [9].

Medical decision support could play a pivotal role in enhancing health care decision-making as it integrates pertinent, organized clinical knowledge and patient data into health-related decisions and processes [12]. Multiple stakeholders, including patients, health care providers, and those involved in patient care, can receive a mix of general clinical insights, patient-specific data, or both. Therefore, a quantitative approach that combines treatment benefits and drawbacks with individual preferences to effectively guide medical decisions could be multicriteria decision-making (MCDM) [13]. MCDM or multicriteria decision analysis (MCDA) is a valuable subdiscipline of operations research, particularly beneficial when dealing with multiple objectives, such as treatment-related outcomes, in benefit-risk analysis [14,15]. A typical MCDM problem consists of 4 key phases: option formulation, criteria selection, criteria weighting, and the decision-making process [16].

### Objective

**Figure 1:**
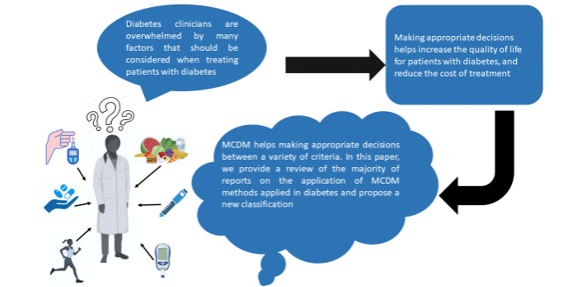
Graphical abstract of the paper. MCDM: multicriteria decision-making.

By considering the abovementioned factors, the primary aim of this research is to assess the use and practicality of MCDM methods in the context of diabetes. Our goal is to examine the various ways in which MCDM techniques have been used to study diabetes and present an innovative categorization of their applications in this field. Figure 1 demonstrates the graphical abstract of the paper.

## Methods

### Search Strategies

A query was carried out on PubMed, Elsevier, Embase, MEDLINE, Scopus, MBC, Springer, IEEE, MDPI, Taylor and Francis Online, and Google Scholar based on published articles. The keywords for our paper were extracted from Medical Subject Headings (MeSH). The keywords “diabetes” and “glucose” were combined with MCDM techniques terms such as TOPSIS, AHP, and multi-criteria-decision-making using the Boolean operator AND/OR. The specific query searched was: ((diabetes OR glucose) AND (AHP OR TOPSIS OR MCDM OR multi-criteria-decision-making)).

### Inclusion and Exclusion Criteria

**Figure 2:**
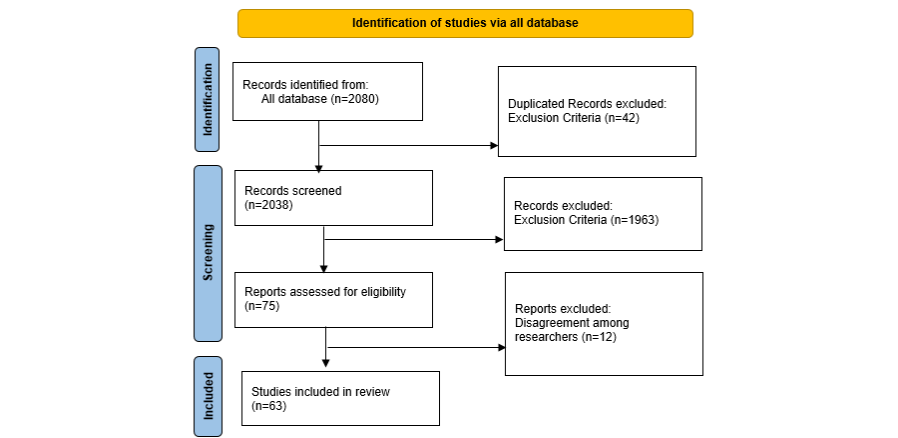
PRISMA (Preferred Reporting Items for Systematic Review and Meta-Analyses) flowchart.

We initially eliminated any duplicate articles from various sources after receiving the results of an initial collection of relevant articles and then manually inspected the remaining articles to assess them under the inclusion criteria. The inclusion criteria were any English papers published between 2003 and 2023. Research, review, conference, and case report articles with an abstract or full text were taken into account. Non-English articles and other research forms, such as letters to editors and brief messages, were excluded. Out of almost 2210 articles, only 63 were found and chosen based on keywords and all of our criteria. The article selection process was based on PRISMA (Preferred Reporting Items for Systematic Review and Meta-Analyses; Figure 2) [17].

## Results

### Overview

Based on Figure 2, after removing duplicates and examining according to the inclusion and exclusion criteria, 63 publications were included in the final evaluation. Based on our investigation to reveal the frequency of publications in databases, it became clear that most of the publications were indexed in Google Scholar, with 60 publications; PubMed, with 17 publications; and Springer and IEEE, with 8 and 7 publications, respectively.

We initially provided a concise overview of MCDM and its techniques, followed by the presentation of our research findings gathered from reviewing publications.

### MCDM Techniques Overview

Since so many choices in our modern lives depend on a multitude of factors, the decision can be made by giving various criteria varying weights, which is done by expert groups. Determining the structure and explicitly evaluating several criteria is crucial. Therefore, constructing and resolving multicriteria planning and decision-making challenges is referred to as MCDM. As a result, MCDM is composed of a set of numerous criteria, a set of alternatives, and some sort of comparison between them [18-20].

No alternative optimizes all criteria uniformly in multicriteria optimization assignments. Any solution to the multicriteria task that enhances a specific criterion can be examined, but the task must ultimately have a preferred option. The decision maker must provide more details to select the best decision. Throughout its brief history of about 50 years, MCDM has been an interesting study topic [20]. There are 2 categories of MCDM approaches: multiattribute decision-making (MADM) and multiobjective decision-making (MODM) [19,20].

**Figure 3:**
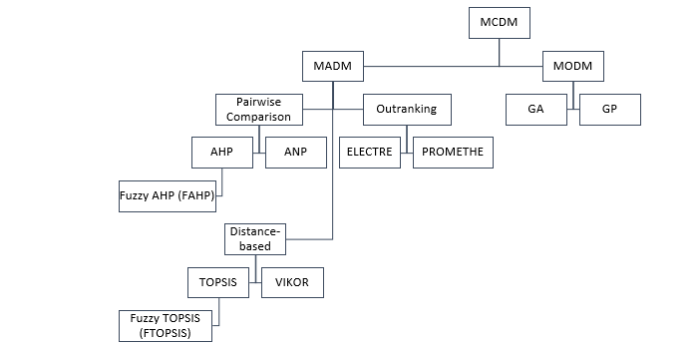
Hierarchical structures of MCDM methods. AHP: analytic hierarchy process; ANP: analytical network process; ELECTRE: Elimination Et Choix Traduisant la Realité; GA: genetic algorithm; GP: goal programming; MADM: multiattribute decision-making; MCDM: multicriteria decision-making; MODM: multiobjective decision-making; PROMETHEE: preference ranking organization method for enrichment of evaluations; TOPSIS: technique for order of preference by similarity to ideal solution; VIKOR: ViseKriterijumska Optimizacija I Kompromisno Resenje.

In order to find the optimal answer, decision makers in MADM choose to categorize, rank, or prioritize a limited number of choices. Pairwise comparison, outranking, and distance-based approaches are the 3 basic methods used in MADM. Pairwise comparison involves evaluating and contrasting the weights of several criteria using a base scale. Analytic hierarchy process (AHP) and analytical network process (ANP) are frequently used in pairwise comparison [21]. Outranking approaches offer a variety of options and determine whether one option has any sort of dominance over the others [22]; instances of outranking techniques include Elimination Et Choix Traduisant la Realité (ELECTRE) and preference ranking organization method for enrichment of evaluations (PROMETHEE) [21]. The solution with the shortest distance to the ideal point is considered the best according to distance-based techniques, which measure the distance a solution is from the ideal point. The technique for order of preference by similarity to ideal solution (TOPSIS) and ViseKriterijumska Optimizacija I Kompromisno Resenje (VIKOR) are 2 popular distance-based methodologies [21]. Unlike MADM, MODM handles situations where there are many decision makers and an infinite number of possibilities. All of these MCDM methods are presented in Figure 3. The most efficient MCDM techniques are introduced in the following sections.

### AHP Method

**Figure 4:**
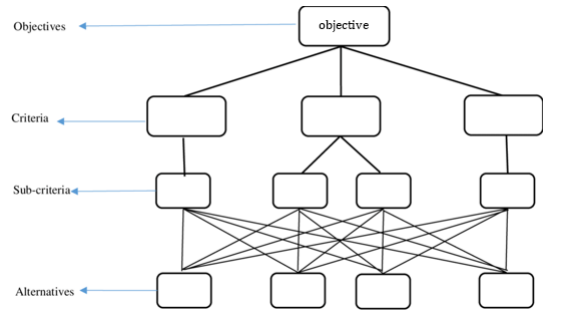
Hierarchical structure of analytic hierarchy process.

Saaty [23] was the first to introduce the AHP. As shown in Figure 4, AHP includes the decision’s objective at the top, the criteria and subcriteria in the middle, and the collection of alternatives at the bottom [7]. The key benefits of AHP are its scalability and ease of usage. AHP can be applied using Excel (Microsoft) or web-based tools such as Transparent Choice, SpiceLogic, Decerns MCDA, MATLAB (MathWorks), R (R Core Team), and Super Decisions.

### TOPSIS Method

**Figure 5:**
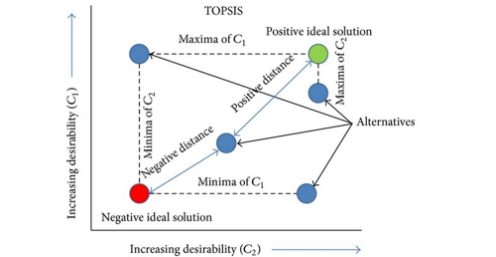
TOPSIS method. TOPSIS: technique for order of preference by similarity to ideal solution.

As shown in Figure 5, TOPSIS is a distance-based technique that Hwang and Yoon [24] proposed in 1981. The TOPSIS technique makes it easy to define the positive and negative ideal solutions by presuming that each criterion tends to monotonically increase or reduce use. A Euclidean distance approach is suggested to assess how closely the alternatives resemble the ideal solution. The preferred order of the alternatives will be determined by a series of comparisons of their relative distances. The general principle behind this approach is that the optimal option should be closest to the ideal solution and the farthest distance from the negative ideal solution. In the ideal solution, the ideal solution has the best attribute values, maximizes the benefit criteria, and minimizes the cost criteria. In the negative ideal solution, the negative solution has the worst attribute values, maximizes the cost criteria, and minimizes the benefit criteria [19,21].

### ANP Method

Due to the inability of AHP to produce an adequate rating with a limited number of possibilities, the majority of organizations do not use it often. Therefore, Saaty [25] suggested ANP as a continuation of AHP. Decision makers are capable of making decisions in difficult situations, according to ANP's capability [21].

### Weighting Methods

One of the crucial phases of MCDM problems is determining the weights of the criterion [26]. Several weighing techniques can be divided into the following groups: (1) subjective weighting method: AHP, Weighted Sum Model (WSM) [27], and Weighted Product Model (WPM) [27]; (2) objective weighting method: Entropy method [28] and Criteria Importance Through Intercriteria Correlation (CRITIC) [28]; and (3) integrated method: step-wise weigh assessment ratio analysis (SWARA) [29] and Weighted Aggregated Sum Product Assessment (WASPAS) [28].

Following a thorough analysis of all of the MCDM publications in the field of diabetes research during a 2-decade period, it was evident that, starting in 2016, the number of publications in this area has been steadily rising, reaching 10 in 2022.

Then, a new classification of the applications of MCDM approaches in diabetes was proposed: (1) selection of diabetes medication, (2) diagnosis of diabetes, (3) meal recommendation for diabetes, (4) diabetes management, (5) diabetes complication, and (6) estimation of diabetes prevalence.

### Selection of Diabetes Medication

Table 1 shows that approximately 30% (n=19/63) of the publications focused on using MCDM techniques to determine the optimal diabetes medication among various options. Notably, AHP and fuzzy AHP, with 6 and 4 mentions, respectively, were the most frequently used methods.

**Table 1:** Diabetes medication publications.

Reference	Methods	Objective	Results
Maruthur et al [14]	AHP^a^	Select oral T2D^b^ medications	Sitagliptin, sulfonylureas, and pioglitazone
Eghbali-Zarch et al [29]	SWARA^c^ method, ratio analysis, and the FMULTIMOORA^d^ method	Choose the pharmacological treatment for T2D	Metformin should be used as the first-line medication, followed by sulfonylurea, glucagon-like peptide-1 receptor agonist, dipeptidyl peptidase-4 inhibitor, and insulin
Eghbali-Zarch et al [28]	WASPAS^e^, entropy, and CRITIC^f^	Determine the final ranking of the medications	Proposed a model to help endocrinologist to choose the best medicine
Zhang et al [30]	TOPSIS^g^	Ranking of diabetes medicines	CDSS^h^ can assist young doctors and nonspecialty physicians with medication prescriptions
Maruthur et al [31]	AHP	Select oral T2D medications	AHP will aid, support, and enhance the ability of decision makers to make evidence-based informed decisions consistent with their values and preferences
Nag and Helal [32]	Fuzzy AHP and AHP	Classification of diabetic medications	Fuzzy AHP model can better handle the ambiguity of decision makers
Chen et al [33]	Entropy	Choose pharmaceuticals	AGI^i^, DPP4^j^, MET^k^, Glinide, SU^l^, and TZD^m^
Wang et al [34]	AHP and ANP^n^	Combine different clinical, economic, and medical decision-making elements	Modifying one's lifestyle, taking metformin, and receiving insulin injections
Bao et al [35]	MCDA^o^	Assess medicine for diabetes	Five DPP4 inhibitors was valuable
Onar and Ibil [36]	Fuzzy AHP	Considered the best oral antidiabetic	Proposed a decision support system
Zhang et al [37]	MCDA	Examine the Mudan Granules	The new medication was acceptable
Cai et al [38]	AHP	Evaluate strains of the efficacy of the LAB^p^ with possible antidiabetic capabilities	Potential antidiabetic effect
Sekar et al [39]	Fuzzy PROMETHEE^q^	Choose the best course of therapy	Giving the high peace of treatment to the most affected people
Mühlbacher et al [40]	AHP and BWS^r^	Evaluate patients’ preferences for various T2D treatment parameters	Proposed a model
Mahat and Ahmad [41]	Fuzzy AHP	Identify and choose the most efficient thermal massage treatment session	Number of therapy sessions (per day) was the most important factor
Pan et al [42]	Fuzzy AHP	Determine the weights of the various physiological factors	The mathematical model of exercise rehabilitation program for patients with diabetes was established
Rani et al [43]	COPRAS^s^	Select T2D medication treatment	Developed a new formula-based PFSs^t^ and evaluated its feasibility by applying the model on selecting the T2D pharmacological therapy
Balubaid and Basheikh [44]	AHP	Developed a mathematical decision-making model that prioritizes the available diabetes medication based on criteria	Metformin, pioglitazone, sitagliptin, and glimepiride were ranked first, second, third, and fourth, respectively
Mühlbacher et al [45]	AHP and BWS	Examine the key patient-related decision criteria involved in the medicinal treatment of T2D	For oral antidiabetes-treated patient groups and insulin-treated patient groups, HbA1c^u^ level, delay of insulin therapy, and occurrence of hypoglycemia were ranked first, second, and third, respectively

^a^AHP: analytic hierarchy process.

^b^T2D: type 2 diabetes.

^c^SWARA: step-wise weigh assessment ratio analysis.

^d^FMULTIMOORA: full multiplicative form.

^e^WASPAS: Weighted Aggregated Sum Product Assessment.

^f^CRITIC: Criteria Importance Through Intercriteria Correlation.

^g^TOPSIS: technique for order of preference by similarity to ideal solution.

^h^CDSS: clinical decision support system.

^i^AGI: α-glucosidase.

^j^DDP4: dipeptidyl peptidase-4.

^k^MET: meglitinide.

^l^SU: sulfonylureas.

^m^TZD: thiazolidinedione.

^n^ANP: analytical network process.

^o^MCDA: multicriteria decision analysis.

^p^LAB: lactic acid bacteria.

^q^PROMETHEE: preference ranking organization method for enrichment of evaluations.

^r^BWS: best–worst-scaling.

^s^COPRAS: Complex Proportional Assessment.

^t^PFS: Pythagorean Fuzzy Set.

^u^HbA1c: hemoglobin A1c.

### Diagnosis of Diabetes

Table 2 displays that roughly 19% (12/63) of the publications centered on the application of MCDM techniques for aiding general practitioners and endocrinologists in diagnosing diabetes. Among these, AHP and TOPSIS were the most commonly cited methods, with 4 and 3 mentions, respectively.

**Table 2:** Diabetes diagnosis publications.

Reference	Methods	Objective	Risk factors	Results
Zulqarnain et al [6]	TOPSIS^a^	Investigate the prevalence of diabetes among women and men	Age, weight, height, BMI, systolic and diastolic BP^b^, urine creatinine, albuminuria, and ACR^c^	Female patients were more likely to develop diabetes
Abdulkareem et al [7]	Fuzzy AHP^d^	Predict diabetes risks	Weakness, obesity, delayed healing, alopecia, muscle stiffness, polydipsia, polyuria, visual blurring, sudden weight loss, and itching	FAHP^e^ model is an excellent tool for diagnosing medical disorders based on many criteria
Abbasi et al [46]	AHP	Identify the most significant risk factors for GDM^f^	A history of GDM or impaired glucose tolerance in previous pregnancies and a history of macrosomia in the infant	N/A^g^
Yas et al [47]	Fuzzy TOPSIS	Identify the symptoms of diabetes	Age, pregnancies, glucose, blood pressure, skin thickness, insulin, BMI, and diabetes pedigree function	Proposed a framework to recognize the symptoms of disease
Amin-Naseri and Neshat [48]	AHP	Determine the likelihood of developing T2D^h^	FBS^i^ index, PRF^j^, BMI, diet, age, BP, gender, family history, and smoking status	DIBAR^k^, a knowledge-based expert system
El-Sappagh et al [49]	Fuzzy AHP	Diagnosis of diabetes	N/A	Created a new, systematically interpretable FRBS^l^ framework
Baha et al [50]	AHP	Diagnosis of diabetes	Heredity, sex, ethnicity, age, impaired glucose tolerance, gestational diabetes, and so forth	Recognized top 3 most important risk factors: heredity, obesity, and physical inactivity
Sharma and Sharma [51]	EDAS^m^	Forecast diabetes	N/A	Combined MCDM^n^ with machine-learning techniques to find the best forecasting model
Malapane et al [52]	WPM^o^	Forecast diabetes	N/A	Combined WPM method with machine learning to select the best model
Felix et al [53]	TOPSIS	Identification of the most important T2D risk factors in the Pima Indian database	Blood glucose, BP, blood cholesterol, obesity, blindness, physical inactivity	Blindness, obesity, and inactivity were the risk factors with greatest impact
Sankar and Jeyaraj [54]	AHP	Forecast diabetes in women	N/A	Propose a model for predicting diabetes among women
Bondor and Mureşan [55]	TOPSIS	Solve the problem of multicollinearity between criteria in diabetes diagnosis	N/A	Proposed a new algorithm which removed the multicollinearity among criteria

^a^TOPSIS: technique for order of preference by similarity to ideal solution.

^b^BP: blood pressure.

^c^ACR: albumin creatinine ratio.

^d^AHP: analytic hierarchy process.

^e^FAHP: fuzzy analytic hierarchy process.

^f^GDM: gestational diabetes mellitus.

^g^N/A: not applicable.

^h^T2D: type 2 diabetes.

^i^FBS: fasting blood sugar.

^j^PRF: physical risk factors.

^k^DIBAR: Created Diabetes Risk Assessment.

^l^FRBS: fuzzy rule-based systems.

^m^EDAS: evaluation based on distance for average solution.

^n^MCDM: multicriteria decision-making.

^o^WPM: Weighted Product Model.

### Meal Recommendation for Diabetes

According to Table 3, a total of 8 (13%) out of 63 publications focused on using MCDM techniques to assist people with diabetes in making the healthiest food choices from their food options, considering factors such as fat content, carbohydrate content, and calorie count. Among these, AHP was mentioned most frequently, with 6 instances.

**Table 3:** Meal recommendation publications.

Reference	Methods	Objective	Criteria	Results
Gaikwad et al [56]	AHP^a^	Recommend a particular ice cream for patients with diabetes	Sugar, cholesterol, dietary fiber, and proteins	Ben & Jerry’s Butter Pecan was enriched with all 4 criteria
Sharawat and Dubey [57]	AHP	Find out the best diet for a patient with diabetes among 3 alternatives: solid food, liquid food, and fluid food	Calories, body fat, healthy carbs, and dietary needs	Solid food was selected as the best
Santoso et al [58]	Fuzzy AHP	Designed a new yogurt product for patients with diabetes	N/A^b^	N/A
Zadeh et al [59]	AHP	Proposed a personalized meal-planning strategy	N/A	Proposed an affordable and culturally appropriate meals that would provide all the nutrition needed for a diabetic while still being mindful of calories and carbs
Gulint and Kadam [60]	AHP and TOPSIS^c^	Recommended shakes and ice cream for patients with diabetes	Sugar, cholesterol, carbs, fat, protein, and dietary fiber	Selected a type of ice cream that satisfies all criteria
Gaikwad et al [61]	ANP^d^	Recommendation of a particular ice cream	Sugar, calories, cholesterol, and proteins	Selected a type of ice cream that satisfies all criteria
Gaikwad et al [62]	AHP	Recommendation of a particular ice cream	N/A	Proposed a model combination of AHP-GA^e^ and AHP-CI^f^ to recommend an ice cream to patients with diabetes
Gaikwad et al [63]	AHP	Recommendation of a particular ice cream	Sugar, protein, cholesterol, and dietary fiber	Patient having a high sugar level of 262 mg/dl can consume an ice cream lower sugar like Breyers butter almond, also patient with low sugar level of 77 mg/dl can consume high sugar ice cream like Breyers

^a^AHP: analytic hierarchy process.

^b^N/A: not applicable.

^c^TOPSIS: technique for order of preference by similarity to ideal solution.

^d^ANP: analytical network process.

^e^AHP-CI: analytic hierarchy process–cohort intelligence.

^f^AHP-GA: analytic hierarchy process–genetic algorithm.

### Diabetes Management

Based on Table 4, additional applications of MCDM techniques, particularly AHP methods, in diabetes management (14/63, 22%) encompass tasks such as identifying ideal locations for diabetes clinics, allocating resources for diabetes care, assessing the current diabetes applications, and constructing models to prioritize criteria that bolster the safety of the insulin supply chain.

**Table 4:** Diabetes management publications.

Reference	Method	Results
Gupta et al [64]	TOPSIS^a^, VIKOR^b^, PROMETHEE II^c^	Assess current mHealth^d^ applications for T2D^e^, including Glucose Buddy, mySugr, Diabetes: M, Blood Glucose Tracker, and OneTouch Reveal
Wang et al [65]	ANP^f^ and CRITIC^g^	Assess the influence of social support on T2DM^h^ self-management
Mishra et al [66]	AHP^i^	Created and used the SCP^j^ assessment methodology for Indian diabetes clinic
Mishra [67]	AHP	Developed a customized service quality assessment model for diabetes care
Mishra [68]	Fuzzy TOPSIS	Proposed 3 alternatives for the placement of a diabetes clinic using the SLP^k^ method
Byun et al [69]	AHP	Improving the treatment compliance of patients with diabetes
Mehrotra and Kim [70]	New multicriterion, robust weighted-sum methodology	Calculate the amount of funding allocated to diabetes preventive initiatives across the United States to reduce the weighted sum of diabetes prevalence and outcomes caused by improper health expenditure
Haji et al [71]	AHP and TOPSIS	Create a model that can prioritize and pick the optimal criterion for optimizing insulin safety
Suka et al [72]	AHP	Described a clinical decision support system that enhance dynamic decision-making
Fico et al [73]	AHP	Selected the best tool for screening and managing T2D
Long and Centor [74]	AHP	Assess the relative significance of 4 frequently used diabetes quality indicators: measuring HbA1c^l^, measuring LDL^m^, performing a dilated eye examination, and performing a foot examination
Gajdoš et al [75]	TOPSIS	Proposed a concept of chronic care management, which could increase effectiveness and reduce the cost of health care provided to patients with T2D
Gupta et al [76]	CODAS-FAHP^n^ and MOORA-FAHP^o^	Assess the usability of mHealth applications to monitor T2D by developing 2 hybrid decision-making methods
Chang et al [77]	Delphi-AHP	Recommended a Delphi-AHP framework to establish agreement in creating a decision-making algorithm for evaluating the balance of benefits and risks associated with the use of complementary and alternative medicine for diabetes

^a^TOPSIS: technique for order of preference by similarity to ideal solution.

^b^VIKOR: ViseKriterijumska Optimizacija I Kompromisno Resenje.

^c^PROMETHEE II: preference ranking organization method for enrichment of evaluation II.

^d^mHealth: mobile health.

^e^T2D: type 2 diabetes.

^f^ANP: analytical network process.

^g^CRITIC: Criteria Importance Through Intercriteria Correlation

^h^T2DM: type 2 diabetes mellitus.

^i^AHP: analytic hierarchy process.

^j^SCP: Supply Chain Partnership.

^k^SLP: Systematic Layout Planning.

^l^HbA1c: hemoglobin A1c.

^m^LDL: low-density lipoprotein.

^n^CODAS-FAHP: combine distance-based assessment-fuzzy AHP.

^o^MOORA-FAHP: multiobjective optimization on the basis of ratio analysis-fuzzy AHP.

### Diabetes Complication

T2D is a significant global public health issue, characterized by 2 categories of harm: macrovascular (involving large arteries) and microvascular (involving small blood vessels). Macrovascular disease such as strokes and microvascular diseases such as retinopathy, nephropathy, and neuropathy [7]. MCDM techniques, especially TOPSIS, as shown in Table 5, are used to assist endocrinologists and general practitioners in analyzing the severity of these complications, forecasting their likelihood of occurrence, and pinpointing the risk factors for them (n=7).

**Table 5:** Diabetes complication diagnosis publications.

Reference	Methods	Objective	Criteria	Complications	Results
Ebrahimi and Ahmadi [78]	Fuzzy TOPSIS^a^	Analyzed the severity caused by diabetes	High cholesterol, high BP^b^, obesity, physical inactivity, smoking, family history, age, and sex	Neuropathy, diabetic retinopathy, cardiovascular disease, kidney disease, foot ulcer, and amputation	Cardiovascular disease was the most important complication in the problem
Ahmadi and Ebrahimi [79]	MCDM^c^	Assessed the severity of difficulties caused by diabetes	Ischemic heart disease, heart failure, heart stroke, ketoacidosis, diabetic ulcer, neuropathy, and lower extremely amputation	Cardiovascular disease, diabetic ketoacidosis, lower extremity complications, and lower extremity amputation	Proposed a new hybrid algorithm that calculate the severity of damage caused by diabetes
Bondor et al [80]	TOPSIS	Identification of the risk factors in kidney disease	Urinary albumin per creatinine ratio and glomerular filtration	Diabetic kidney	Rank the risk factors of microalbuminuria and eGFR^d^ to evaluate the risk factor for CKD^e^
Ahmed et al [81]	TOPSIS and entropy	Detection of DR^f^ through machine learning and TOPSIS models	Criteria of TOPSIS model: AUC^g^, accuracy, precision, F1-score, recall, TPR^h^, FNR^i^, FPR^j^, TNR^k^, and time	DR	According to TOPSIS, Adaboost model ranks at the best model to detect DR
Bondor et al [82]	VIKOR^l^	Rank risk factors of diabetic kidney disease	Serum adiponectin, triglycerides, SBP, duration of diabetes and age, Malondialdehyde, and HDL^m^-cholesterol	Diabetic kidney	Identification of diabetic kidney disease risk factors
Alassery et al [83]	Fuzzy AHP^n^ and Fuzzy TOPSIS	Determine the impact of mental health in patients with diabetes	BMI, SBP, DBP^o^, age, height, exercise	Mental health	The model showed the applicability and impact of mental health in patients with diabetes
Wang et al [84]	AHP	Relieve the pain in patients with diabetes	N/A^p^	Diabetic neuropathy and foot ulcers	Selection of shoe lasts for footwear design to help relieve the pain associated with diabetic neuropathy and foot ulcers

^a^TOPSIS: technique for order of preference by similarity to ideal solution.

^b^BP: blood pressure.

^c^MCDM: multicriteria decision-making.

^d^GFR: estimated glomerular filtration rate.

^e^CKD: chronic kidney disease.

^f^DR: diabetic retinopathy.

^g^AUC: area under the curve.

^h^TPR: true positive rate.

^i^FNR: false negative rate.

^j^FPR: false positive rate.

^k^TNR: true negative rate.

^l^VIKOR: ViseKriterijumska Optimizacija I Kompromisno Resenje.

^m^HDL: high-density lipoprotein.

^n^AHP: analytic hierarchy process.

^o^DBP: diastolic blood pressure.

^p^N/A: not applicable.

## Discussion

### Principal Findings

Given the multitude of choices involved in selecting diabetes medication, meal planning, nutrient intake, diabetes management apps, and speedy diagnosis, endocrinologists, general practitioners, and individuals with diabetes, along with their caregivers, need guidance to make informed decisions. MCDM is a quantitative approach that effectively integrates treatment benefits and drawbacks, as well as individual preferences, to facilitate sound medical decision-making in these complex situations. Consequently, we embarked on an evaluation of the effectiveness of MCDM methods in the context of diabetes.

Based on a notable upward trend in publications within the realm of using MCDM methods in diabetes research over the last 2 decades, this underscores the growing interest among researchers in applying MCDM methods to address diabetes-related challenges. Furthermore, the majority of these publications (n=19) focus on diabetes treatment selection [14,28-45]. Diabetes management (n=14), diagnosis of diabetes (n=12), meal recommendation (n=8), diabetes complications (n=7), and global estimation (n=3) are in the later ranks. This outcome highlights the efficacy of using MCDM methods in the process of choosing diabetes medications.

All MCDM methods in diabetes are classified into 13 groups. AHP is ranked first, having been used in 25 articles. AHP is designed to help individuals and groups make complex decisions by breaking them into a hierarchical structure, comparing and weighting criteria and alternatives, and deriving a rational choice based on these comparisons [7,85,24]. AHP can be applied to diabetes issues and decision-making in several ways including treatment selection [14,31,32,34,36,38-42,44,45], diabetes diagnosis [46,48-50,54], dietary planning [56-60,62,63], diabetes management [66,67,69,71-74,77], complication diagnosis [84], and estimating diabetes prevalence [4,5]. TOPSIS and fuzzy AHP with 9 and 8 publications are in the next ranks, respectively.

As observed, 6 distinct weighting algorithms were recognized, with the Entropy approach ranking highest. The final component in our proposed classification pertains to estimating diabetes prevalence. In a 2013 study, researchers used logistic regression and AHP techniques to produce smoothed age-specific occurrence estimates for adults aged 20 to 79 years. These estimates were then used to calculate population projections for the years 2013 and 2035, foreseeing an increase in the number of individuals with diabetes to 592 million by 2035 [4]. In another investigation conducted by the IDF in 2015, AHP and logistic regression methods were used to estimate that there were 415 million people (ranging from 340 million to 536 million) with diabetes. Projections indicate that this figure is expected to reach 642 million (ranging from 521 million to 829 million) by 2040 [5].

### Conclusions

One of the most serious health problems of the 21st century, whose prevalence is rapidly increasing, is diabetes mellitus. Almost all areas of diabetes research have seen significant progress to date, particularly in the areas of medication selection, meal selection, diabetes management applications, use of continuous glucose monitoring, and closed-loop system. The advancement of technology has expanded the scope of decision-making responsibilities for general practitioners in the initial stages of patient care. Determining the most optimal choice among numerous options falls within the domain of MCDM.

In this research, for the first time, we reviewed the majority of MCDM papers for diabetes and considered 2 important issues in the field of diabetes: examining the usability of MCDM techniques in diabetes and proposing a new classification of applications of MCDM methods in diabetes. Our study highlights that the use of MCDM techniques extends beyond the realm of diabetes medication selection. These methods hold promise for diverse applications, spanning meal planning, diabetes diagnosis, and addressing diabetes-related challenges. This includes tasks such as selecting optimal diabetes management applications from a wide range of options, identifying ideal locations for diabetes clinics, and efficiently allocating resources for diabetes care. Moreover, the analysis reveals that AHP is the preferred and widely embraced strategy and approach, primarily owing to its straightforward structure and user-friendliness. We firmly believe that the adoption of MCDM approaches offers advantages to a broad spectrum of stakeholders, including patients with diabetes, endocrinologists, general practitioners, caregivers, and health care policy makers. These techniques have the potential to serve as valuable tools for general practitioners, assisting in quicker diabetes diagnosis and more accurate medication selection, ultimately reducing patient costs and lifestyle concerns.

## Appendix

Multimedia Appendix 1 PRISMA checklist.

## Abbreviations

ADA: American Diabetes Association
AHP: analytic hierarchy process
ANP: analytical network process
CRITIC: Criteria Importance Through Intercriteria Correlation
ELECTRE: Elimination Et Choix Traduisant la Realité
IDF: International Diabetes Federation
MADM: multiattribute decision-making
MCDA: multicriteria decision-analysis
MCDM: multicriteria decision-making
MeSH: Medical Subject Headings
MODM: multiobjective decision-making
PRISMA: Preferred Reporting Items for Systematic Review and Meta-Analyses
PROMETHEE: preference ranking organization method for enrichment of evaluations
SWARA: step-wise weigh assessment ratio analysis
TOPSIS: technique for order of preference by similarity to ideal solution
T2D: type 2 diabetes
VIKOR: ViseKriterijumska Optimizacija I Kompromisno Resenje
WASPAS: Weighted Aggregated Sum Product Assessment
WPM: Weighted Product Model
WSM: Weighted Sum Model
